# The Vector-Borne Zoonotic Nematode *Thelazia callipaeda* in the Eastern Part of Europe, with a Clinical Case Report in a Dog in Poland

**DOI:** 10.3390/pathogens10010055

**Published:** 2021-01-09

**Authors:** Leszek Rolbiecki, Joanna N. Izdebska, Marta Franke, Lech Iliszko, Sławomira Fryderyk

**Affiliations:** 1 Department of Invertebrate Zoology and Parasitology, Faculty of Biology, University of Gdańsk, Wita Stwosza 59, 80-308 Gdańsk, Poland; biojni@ug.edu.pl (J.N.I.); slawomira.fryderyk@ug.edu.pl (S.F.); 2 Veterinary Office, Lutowiska 68, 38-713 Lutowiska, Poland; marta.franke@onet.pl; 3 Department of Vertebrate Ecology and Zoology, Faculty of Biology, University of Gdańsk, Wita Stwosza 59, 80-308 Gdańsk, Poland; leiliszko@gmail.com

**Keywords:** *Thelazia callipaeda*, eyeworm, zoonotic nematode, thelaziosis, dog, Poland, Eastern Europe

## Abstract

*Thelazia callipaeda* is a zoonotic nematode transmitted by drosophilid flies. It causes ocular thelaziosis, a disease of carnivores, such as dogs, cats, and foxes, and also humans. The parasite has thus far been observed in various areas of Eurasia, including 20 countries within Europe. The present study documents its presence in the south-east region of Poland, near the Ukraine border. An adult nematode was removed from the conjunctival sac of a dog showing ocular inflammation and purulent discharge. The dog’s precise origin is unknown. Based on its localization and morphometrical features, the nematode was identified as a *Thelazia callipaeda* adult male. The present study is the first report of *T. callipaeda* in a dog in Poland.

## 1. Introduction

*Thelazia callipaeda* Railliet et Henry, 1910 (Spirurida, Thelaziidae) is a parasitic nematode of the eye known to cause thelaziosis, a parasite infection of carnivores, lagomorphs and humans [1,2]. The infection can manifest with conjunctivitis, epiphora, visual disturbances and even keratitis, and corneal ulceration (e.g., [3,4]). The parasite was first discovered over one hundred years ago in Punjab, India [5], and has been thus far observed in other areas of Asia, such as China, Myanmar, India, Indonesia, Thailand, Taiwan, South of Korea, Japan, Bangladesh, Nepal, Vietnam, and the former USSR [3,6,7,8,9,10,11,12,13,14], and in 20 countries in Europe [15,16]. Despite its wide area of distribution and significant pathogenic (including zoonotic) importance, the parasite has been identified only relatively rarely and in few localities; however, its distribution is potentially far greater, particularly since data obtained from veterinary practices are not always included in academic studies.

Previous records from Western, Central and Southern Europe suggest that the nematode may also occur in neighboring countries, such as Poland and Ukraine. The present study is the first report of *T. callipaeda* in a dog in Poland.

## 2. Results

### 2.1. Case Report

**Figure 1:**
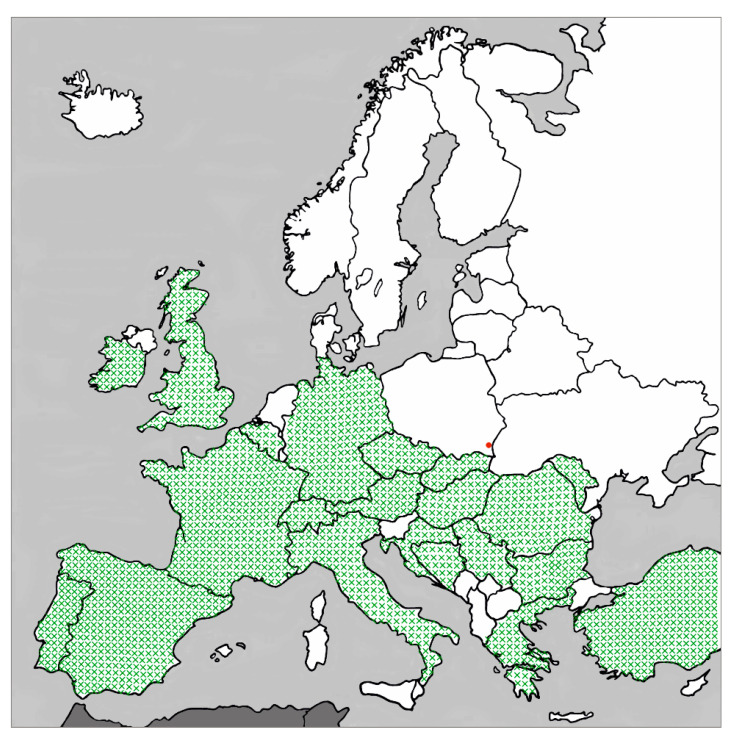
Geographical distribution of *Thelazia callipaeda* in Europe and locality (Krywe) of the present report in a dog in Poland.

A stray dog was observed in Poland, in the border area with Ukraine (approx. 5 km from the border), from July 2019 (Figure 1). The dog’s precise origin is unknown. The detailed fate of the dog in the period from the first to the second observation is also unknown. In November 2019, after adoption, the dog was subjected to a veterinary examination and the necessary treatments—castration, deworming, vaccinations, and treatment of skin mycosis. During the first veterinary examination, an inflammation, erythema and excessive purulence of both eyes, particularly in one eye, were also diagnosed. The dog was in general poor condition, thus, the ocular symptoms were not then linked to the presence of parasites. He was initially treated with eye drops containing antibiotic tobramycin and corticosteroid dexamethasone (Tobradex^®^, S.A. ALCON-COUVREUR N.V., Belgium) for three weeks, but with no effect. At the subsequent examination, a nematode was found in one of the eyes. No worms were found in the other eye. Symptoms and eye irritation resolved several days after removal of the nematode and the administration of an antiparasitic drug composed of imidacloprid and moxidectin (Advocate^®^, KVP Pharma + Veterinär Produkte GmbH, Kiel, Germany).

### 2.2. Description of the Nematode

**Figure 2:**
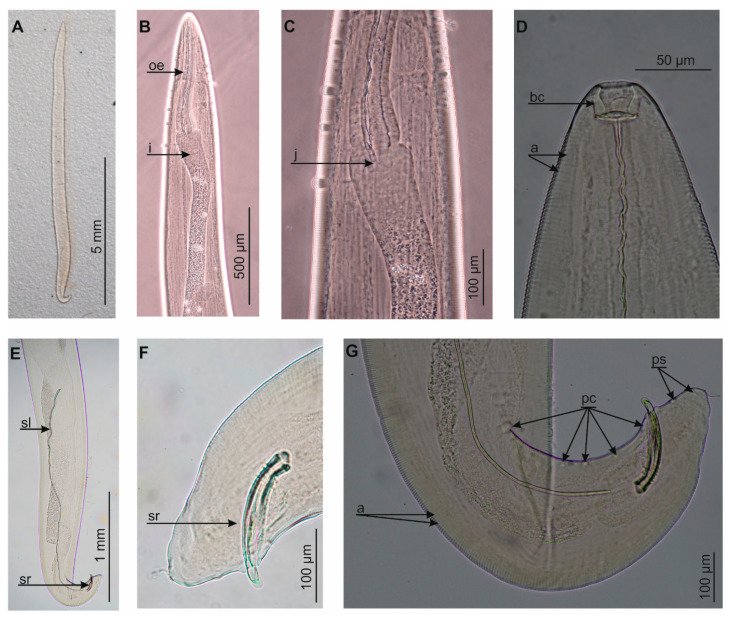
*Thelazia callipaeda*, (**A**) total view, (**B**) anterior end, (**C**) junction between oesophagus and intestine, (**D**) cephalic end, (**E**) posterior end, (**F**) tail, (**G**) tail and papillae; a: annellations, bc: buccal capsule, i: intestine, j: junction, oe: oesophagus, pc: pre-cloacal papillae, ps: post-cloacal papillae, sl: left spicule, sr: right spicule.

The isolated nematode (Figure 2) had a body length of 9.5 mm and a maximum width of 450 μm, while the width at the level of the oesophagus-intestinal junction was 265 μm. The caudal end was ventrally curved with pre-cloacal and post-cloacal papillae. The nematode had a clearly serrated cuticle, except for the buccal capsule. The buccal capsule was hexagonal, 25 μm long and 30 μm wide. The oesophagus was 580 μm in length, while its width was 92.5 μm at the posterior end and 60 μm at the anterior end. The tail was 90 μm long and showed two spicules; the left spicule was 1.88 mm long and the right spicule was 170 mm long. Based on its localization and morpho-anatomical and metrical features, the nematode was identified as a *T. callipaeda* adult male (Figure 2).

## 3. Discussion

In recent years, an increased number of *T. callipaeda* infection clinical cases have been observed throughout Europe (Figure 1) [4,15]. Carnivores are considered the main definitive hosts of *T. callipaeda*, including domestic carnivores, such as domestic dogs or domestic cats [17,18,19,20], or wild carnivores, such as the red fox *Vulpes vulpes* (Linnaeus, 1758), the European badger *Meles meles* (Linnaeus, 1758), the beech marten *Martes foina* (Erxleben, 1777), the Eurasian lynx *Lynx lynx* (Linnaeus, 1758), the golden jackals *Canis aureus* Linnaeus, 1758, the gray wolf *Canis lupus* Linnaeus, 1758, the racoon dog *Nyctereutes procyonoides* (Gray, 1834), the sable *Martes zibellina* (Linnaeus, 1758), the wild cats *Felis silvestris* Schreber, 1777, and the Asiatic black bear *Ursus thibetanus* G. [Baron] Cuvier, 1823 [21,22,23,24,25,26]. However, *T. callipaeda* has been reported also in other mammals, including the European rabbit *Oryctolagus cuniculus* (Linnaeus, 1758), the brown hare *Lepus europaeus* Pallas, 1778 [27,28] and, above all, in humans [1,3,4,11,14]. It is likely that the entire host range has not yet been discovered. It is also possible that under favorable circumstances, *T. callipaeda* may be able to increase its host spectrum and continue its spread by colonizing other less typical hosts. A particularly important consideration in this regard is its zoonotic significance and its increasing incidence in humans [4,29,30].

Thus far, the only known intermediate hosts of *T. callipaeda* are the zoophilic flies of the subfamily Stegamininae (Diptera: Drosophilidae), i.e., *Phortica variegata* (Fallén, 1823) in Europe and *P. kappa* (Máca, 1977), *P. magna* (Okada, 1960), and *P. okadai* (Máca, 1977) in some parts of Asia (summarized in [31,32,33]). According to Huang et al. [34], *P. kappa* is found in Japan, *P. magna* in China and Japan, and *P. okadai* in China, Japan, Korea, and Russia. *Thelazia callipaeda* was first described in Asia at the beginning of the 20th century but has been recorded in European countries since the 1980s [35].

In Europe, *P. variegata* is widely distributed from southern areas up to Scandinavia and from the western to the European part of Russia, including Primorsky Krai [36]. This fly, under the former genus name *Amiota variegata,* was also indicated as the vector of *T. callipaeda* in Japan, but systematic revisions verified the status of the species as being present in Europe and the Caucasus [31,32,33]. In addition, *T. callipaeda* has been identified in some South-East Asia countries (e.g., India, Indonesia), where none of the four *Phortica* species known to act as its vectors could be found. Nevertheless, other *Phortica* species are present in this area, indicating that they may also act as vectors for *T. callipaeda* [32]. In general, the mode of transmission of these nematodes requires a considerable amount of research, as little is known of the flies representing this family and species identification is problematic [34]. However, it is significant that access to an intermediate host does not seem to limit its distribution across the whole of Eurasia.

Therefore, it has been predicted that Poland lies within the range of *T. callipaeda* [37]. Undoubtedly, an indication for this were the findings of *T. callipaeda* in the neighboring countries, i.e., Germany, the Czech Republic, and Slovakia [15] (Figure 1). Out of four records from Germany, two describe cases that are probably introduced [15]. However, cases described from Slovakia [38,39] and from the Czech Republic [40] are autochthonous. A recent finding from the Czech Republic (Prague) is considered the northernmost record of autochthonous canine thelaziosis in Europe [40]. In turn, in Poland, thus far, no comprehensively documented records are available. The only relevant data concerned a case in north-west Poland, probably introduced from Germany, which was presented at a national conference [41], but no extensive data in the form of a reviewed publication has so far been published.

Our present data, documented with helminthological identification and deposition of the material in an academic collection, originate from an area of south-east Poland, 5 km from the border with Ukraine. Disease symptoms observed in the dog (inflammation, erythema, and abundant purulent ocular discharge) were typical for *T. callipaeda* infection and quite severe. In fact, *T. callipaeda* adults and larval stages may cause lacrimation, conjunctivitis, and mild mucopurulent discharge [3,42], with the severity and course depending on the intensity of infection or the presence of secondary bacterial infections [43]. Perhaps the high severity of symptoms was influenced by the general poor condition of the dog, as the intensity of the infection was low. Similar changes with a purulent discharge, conjunctival hyperaemia, were observed in dogs with a much higher level of infection [39]. As such national borders do not serve as barriers for parasites and their hosts, it is difficult to ascertain whether infection occurred in Poland, Ukraine, or in a third country. Nevertheless, our confirmation of the presence of *T. callipaeda* in this part of Europe is significant. According to previous studies, *P. variegata* is found throughout Europe, with the exception of its northern margin [44]. Its presence has been confirmed in Poland [45], although Drosophilidae are considered to be very poorly studied in the country, and their distribution, abundances, population dynamics, or seasonal dynamics have not been investigated in great detail so far. Undoubtedly, this constitutes a limitation to the predictions concerning the potential possibilities of *T. callipaeda* infection in different areas and seasons. Climatic factors may constitute a limiting parameter for the distribution of flies of this genus, and consequently of *T. callipaeda*, because they exhibit the preference for areas and seasons characterized by high temperatures [46]. However, the ecological preferences of *P. variegata* have not yet been studied in detail (e.g., in Poland [45]). Research was done locally, for instance in Italy or other selected regions of Europe, which made it possible to forecast potential distribution [31,32,44]. However, such data are difficult to refer to other regions of Europe characterized by different types of habitats or specific microclimatic factors. For sure, this requires detailed faunistic analyses by taking into account the specificity of local ecosystems. At the same time, information on the distribution of *P. variegata* is typically referred to the cases of mainly canine and human thelaziosis, based on singular observations and on limited areas. The disease is typically referred to rural areas, herding and hunting dogs, or other dogs with a great freedom of movement, which often have the access to areas constituting the natural habitat of *P. variegata* [32]. Without a doubt, the ongoing climate warming, as well as the host migration possibilities, which are difficult to control, may contribute to further records of *T. callipaeda* in other parts of Europe. Based on the presence of typical hosts in the fauna, it can be expected that this nematode has a wider distribution or its dispersion can be predicted. *Phortica variegata* was recorded also in Belarus [47], or in northern Europe, in Norway [48]. Undoubtedly, the growth and expansion of domestic predatory mammal populations, especially dogs whose global population is currently estimated at 900 million [49], can be one of the causes for the expansion of *T. callipaeda* distribution. They can cover large distances thanks to humans, as pets, but also locally, especially feral and stray dogs and other dogs with high freedom of movement. Another cause is the changes in the behavior of certain wild animals, such as fox synurbization.

These nematodes are typically detected based on the symptoms in their hosts, particularly the most commonly studied ones, i.e., domestic mammals. Therefore, a combination of comprehensive study and the monitoring of confirmed and potential intermediate hosts is needed to obtain a fuller picture of the current distribution and population dynamics of *T. callipaeda*. The present report of *T. callipaeda* in a dog in Poland stimulates further research studies in this area, which should include both domestic and wild animals.

## 4. Materials and Methods

### 4.1. Description of the Host

A stray dog named *Smolnik*, male, age ~2 years, weight ~45 kg, was sighted near the Orthodox church in Smolnik village (49°12′55.4″ N, 22°41′45.1″ E). *Smolnik* had a Shepherd phenotype, similar to the Romanian Mioritic Shepherd breed. He was observed in Poland from around July 2019; he probably came from Ukraine. In November 2019, the dog was adopted by a resident of the Krywe village (49°15′04.0″ N, 22°31′12.2″ E, Figure 1) and underwent veterinary examinations and treatments.

### 4.2. Parasitological Analysis

On 20 May 2020, the parasite was removed. The procedure was performed in a veterinary clinic with the dog under general anesthesia (dexmedetomidine and butorphanol) (Lutowiska, Poland). One nematode was removed from the conjunctival sac and fixed in 4% formaldehyde solution for study.

The nematode was placed in polyvinyl-lactophenol solution and identified to species level [1] using phase-contrast microscopy (Nikon Eclipse 50i). All measurements are in micrometers unless otherwise noted. Following analysis, the microscope slide was added to the scientific collections within the framework of the Collection of Extant Invertebrates in the University of Gdańsk, Department of Invertebrate Zoology and Parasitology, Poland.
